# EMOTIONS AND WELL-BEING IN LATER LIFE CHILDHOOD HAPPINESS, SELF-MASTERY, AND LATER-LIFE HEALTH

**DOI:** 10.1093/geroni/igz038.2135

**Published:** 2019-11-08

**Authors:** Haena Lee, Markus H Schafer

**Affiliations:** 1 University of Michigan, Ann Arbor, Michigan, United States; 2 University of Toronto, Toronto, Ontario, Canada

## Abstract

Considerable work has documented that positive childhood memories, especially childhood happiness, predict better health among young adults. However, it is not known whether growing up happy has enduring health consequences across the life course. Using two waves of the National Social Life, Health and Aging Project (2010-2011 and 2015-2016; N = 1,937), we investigate the relationship between childhood happiness and changes in physical, mental, and biological functioning in later life. Childhood happiness was retrospectively assessed using a question: “When I was growing up, my family life was always happy.” Self-rated health, depressive symptoms, and frailty over a five-year period were examined to reflect changes in functional status. Childhood SES and living arrangement were examined to assess childhood sociodemographic background. Educational attainment, family support and strain, and self-mastery were considered as potential mediators. We find that, among other childhood factors, childhood happiness significantly predicts older adult health. Specifically, childhood happiness was associated with better self-rated health and lower depressive symptoms at follow-up, net of baseline health conditions. We did not find a relationship between frailty and childhood happiness. Unlike prior work, we found no significant effect of childhood SES on the measured outcomes. Associations between childhood happiness and self-rated health and depression were mediated by psychosocial resources including self-mastery and perceived social support from family members. This implies that growing up in nurturing, cherished family environment has the potential to cultivate social relationships and build resilience which could provide an important pathway to successful aging.

